# SMDB: pivotal somatic sequence alterations reprogramming regulatory cascades

**DOI:** 10.1093/narcan/zcaa030

**Published:** 2020-10-13

**Authors:** Limin Jiang, Mingrui Duan, Fei Guo, Jijun Tang, Olufunmilola Oybamiji, Hui Yu, Scott Ness, Ying-Yong Zhao, Peng Mao, Yan Guo

**Affiliations:** Comprehensive Cancer Center, Department of Internal Medicine, University of New Mexico, Albuquerque, NM 87109, USA; Comprehensive Cancer Center, Department of Internal Medicine, University of New Mexico, Albuquerque, NM 87109, USA; School of Computer Science and Technology, College of Intelligence and Computing, Tianjin University, Tianjin 300350, China; Department of Computer Science, University of South Carolina, Columbia, SC 29208, USA; Comprehensive Cancer Center, Department of Internal Medicine, University of New Mexico, Albuquerque, NM 87109, USA; Comprehensive Cancer Center, Department of Internal Medicine, University of New Mexico, Albuquerque, NM 87109, USA; Comprehensive Cancer Center, Department of Internal Medicine, University of New Mexico, Albuquerque, NM 87109, USA; Key Laboratory of Resource Biology and Biotechnology in Western China, School of Life Sciences, Northwest University, Xi’an, Shaanxi 710069, China; Comprehensive Cancer Center, Department of Internal Medicine, University of New Mexico, Albuquerque, NM 87109, USA; Comprehensive Cancer Center, Department of Internal Medicine, University of New Mexico, Albuquerque, NM 87109, USA

## Abstract

Binding motifs for transcription factors, RNA-binding proteins, microRNAs (miRNAs), etc. are vital for proper gene transcription and translation regulation. Sequence alteration mechanisms including single nucleotide mutations, insertion, deletion, RNA editing and single nucleotide polymorphism can lead to gains and losses of binding motifs; such consequentially emerged or vanished binding motifs are termed ‘somatic motifs’ by us. Somatic motifs have been studied sporadically but have never been curated into a comprehensive resource. By analyzing various types of sequence altering data from large consortiums, we successfully identified millions of somatic motifs, including those for important transcription factors, RNA-binding proteins, miRNA seeds and miRNA–mRNA 3′-UTR target motifs. While a few of these somatic motifs have been well studied, our results contain many novel somatic motifs that occur at high frequency and are thus likely to cause important biological repercussions. Genes targeted by these altered motifs are excellent candidates for further mechanism studies. Here, we present the first database that hosts millions of somatic motifs ascribed to a variety of sequence alteration mechanisms.

## INTRODUCTION

Somatic mutations occurring in life-supporting protein-encoding genes can cause severe adverse effects on human health (1). Non-coding somatic mutations can also have dreadful consequences (2). Non-coding somatic mutations become especially damaging if they alter *cis*-elements for regulator molecules, including transcription factors (TFs) (3), RNA-binding proteins (RBPs) (4), microRNA (miRNA) seeds (5), miRNA–mRNA 3′-UTR targeting factors (6), etc. We term the consequentially emerged or vanished binding motifs of important regulator molecules as ‘somatic motifs’.

TF motifs are an indispensable element for fueling the transcription machinery; thus, TF somatic motifs attract the most intensive research attention. One extensively analyzed TF binding mutation in human cancers is located in the *TERT* promoter region, which creates a new canonical binding motif TTCCGG for oncogenic E26 transformation-specific (*ETS*) factors; this gained motif allows ETS proteins to bind to the mutated promoter to trigger *TERT* expression, resulting in uncontrolled cell proliferation and eventual tumorigenesis (7).

RBPs bind to the double- or single-stranded RNA in cells through recognizing specific RNA recognition motifs. RBPs have been found to play important roles in the post-transcriptional gene regulation process, and their impact on cancer biology has been well documented (8). Henceforth, the binding motifs of RBPs, primarily located in 3′-UTRs and introns, become another major source of somatic motifs.

miRNAs are a type of small non-coding RNAs, known for their ability to regulate protein-coding genes via complementary binding to the seed regions (six to eight nucleotides from the 5′ end of miRNAs). Somatic mutations in the seed sequences can cause significant deviation from normal miRNA–mRNA regulation networks. For example, it has been shown that mutations in the seed region of miR-96 are responsible for non-syndromic progressive hearing loss (9). Similarly, a mutation in the seed region of miR-84 causes EDICT syndrome (10). miRNA typically binds to 3′-UTR of mRNA (6). A somatic mutation in the 3′-UTR binding region can also disrupt normal miRNA–mRNA binding. Substantial efforts have been made to curate and document somatic mutations in miRNAs and their impact, such as the SomamiR database (5).

We extend the source of somatic motifs from somatic mutations to two additional sequence alteration mechanisms: RNA editing and germline inherited single nucleotide polymorphisms (SNPs). RNA editing is an enzymatic modification process that alters RNA molecule’s nucleotide sequence in relation to the corresponding DNA sequence. RNA editing events mainly come in the form of adenine-to-inosine (A-to-I) and cytosine-to-uracil (C-to-U) substitutions, with the former taking an overwhelming (>90%) dominance (11). Recent studies have revealed significant functional effects of A-to-I RNA editing. Peng *et al.* demonstrated experimentally that non-synonymous A-to-I RNA editing can result in altered protein sequences by modifying amino acids in cancer (12), and may subsequently affect drug sensitivity (13). Furthermore, increased RNA editing activity has been associated with poor cancer prognosis (14).

Even though SNPs are not considered somatic mutations, SNPs can affect disease risk and regulate gene expression as evidenced by the thousands of genome-wide association studies and gene expression quantitative trait loci (eQTL) studies. It has been suggested previously that eQTLs regulate gene expression by affecting TF motifs (15), which indicates that SNPs can affect the binding efficiency if one of the two alleles creates a new binding motif. We hypothesized that SNPs may also affect the binding efficiency of RBPs and miRNAs with similar mechanisms, which can be used to explain a large portion of the *cis*-eQTL regulation mechanism.

As we articulated above, somatic motifs can be ascribed to a variety of sequence alteration mechanisms and they may take place in motifs of all kinds of molecular regulators. Somatic motifs have been studied sporadically with a focus on high potential targets such as *TERT* promoter mutations (7,16). However, somatic motifs have not been curated into a comprehensive resource. Furthermore, previous studies were usually focused on single nucleotide mutations, but have not yet analyzed insertions and deletions sufficiently. In this work, by analyzing multi-omic data of over 30 000 subjects from several large consortiums, we identified millions of somatic motifs ascribed to a variety of sequence alteration mechanisms in relation to TFs, RBPs and miRNAs. These somatic motifs, including a large portion of novel ones, were compiled into somatic motif database (SMDB) for easy searching, browsing and downloading by the research community at large.

## MATERIALS AND METHODS

### Somatic motif detection algorithm

**Figure 1.**
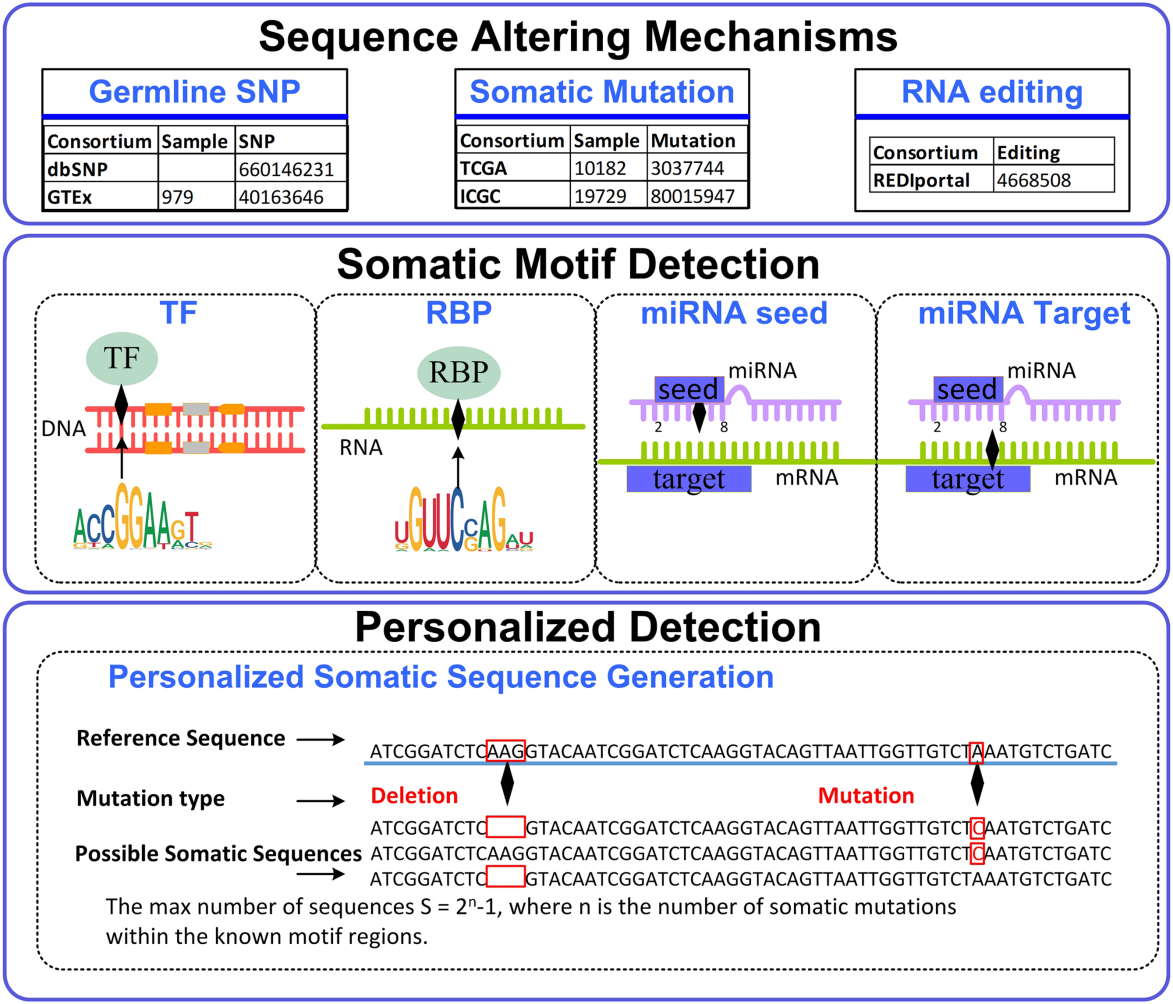
Overview of our somatic motif detection algorithm. Top: A simple scenario to show how somatic sequences are generated based on mutations. Middle: The possible target types of somatic motif. Bottom: Our somatic motif algorithm allows personalized motif search accounting for adjacent single mutations or insertions/deletions (INDELs).

Binding sequence alteration due to either somatic mutations or RNA editing, or any nucleotide modification process can result in disease-promoting biological chain reactions. The overall algorithm of somatic motif detection algorithm is explained in Figure 1. Given a set of somatic mutations, and a list of target motifs (short nucleotide sequences), we detected whether somatic motifs are gained or lost as a consequence of the specified somatic mutations. The foremost step of our algorithm is to derive altered sequences that embed the provided somatic mutations. When somatic mutations occur in proximity to each other, the combinations of *n* nearby mutations lead to 2^*n*^ − 1 alternative somatic sequences that are existent in equal likelihood. Our somatic motif algorithm is capable of handling both small insertions and deletions. Both forward and reverse strands were considered for somatic motif identification.

### Data acquisition

Two sets of somatic mutation data were downloaded. The first set contains 10 182 subjects of 33 cancer types from The Cancer Genome Atlas (TCGA). The second set contains 19 729 subjects of 57 cancer types from 81 projects within the International Cancer Genome Consortium (ICGC). From REDIportal (17), we downloaded 4 668 508 A-to-I RNA editing events. SNP data from dbSNP (v152) of 660 146 174 SNPs were downloaded from NCBI. Furthermore, 40 163 646 *cis*-eQTLs from 49 tissue sites were downloaded from Genotype-Tissue Expression (GTEx). All result sequences are presented in 5′ to 3′ orientation regardless of strand orientation and in the GRCh38 human reference genome.

Seven hundred forty-six transcription binding motifs from the JASPAR database (18) were extracted and primary binding sequences from these 746 motifs were used to detect somatic motifs. Furthermore, human miRNA seed region files were prepared from miRNA information downloaded from miRBase (19). miRNA seed binding mRNA target sequences were obtained from starBase 2.0 (20). Moreover, a total of 3524 RBP binding motif sequences were downloaded from four databases: ATtRACT (2883) (21), oRNAment (454) (22), RBPDB (95) (23) and RBPmap (92) (24). We limited the RBP motif length to >5 nucleotides to reduce potential ambiguity.

## RESULTS AND DISCUSSION

### Web design and interface

**Figure 2.**
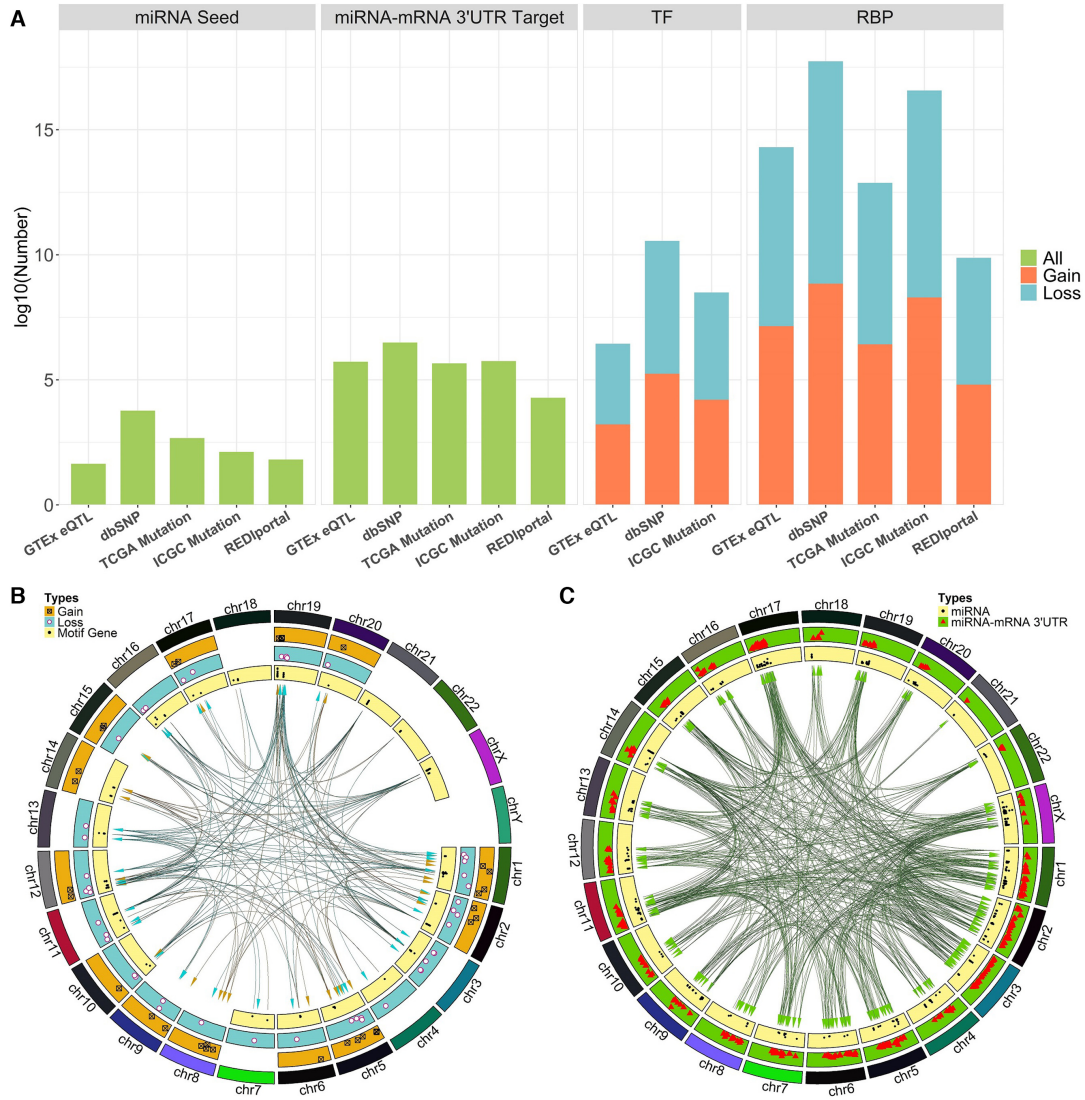
(**A**) The overall results from conducting somatic motif analysis from five data sources (TCGA, ICGC, GTEx, dbSNP, REDIportal) against multiple data sources. The bar represents the log_10_ value of identified somatic motifs. (**B**) Genome-wide visualization of the TF somatic motifs in circos plot. There are four layers in this circos plot. The outer layer represents the genome by chromosome; the second layer (gold) represents somatic mutations that cause gains of TF motif; the third layer (dark slate gray) denotes somatic mutations that caused loss of TF motifs; and the fourth and inner layer (khaki) denotes the location of the binding motif gene. In the middle, the arrows’ color matches the layer’s color and is connecting the proper sequence altering mechanism on the second and third layers to its binding motif gene on the fourth layer. (**C**) Genome-wide visualization of miRNA–mRNA 3′-UTR binding somatic motifs. There are three layers in this circos plot. The outer layer represents the genome by chromosome; the second layer (chartreuse) represents the miRNA–mRNA 3′-UTR binding location; and the third and inner layer (khaki) represents miRNA locations. In the middle, the arrows’ color matches the layer’s color and is connecting the proper sequence altering mechanism on the second layer to its binding miRNA on the third layer.

Through our intense analysis of omic data from several large consortiums, we identified millions of known and novel somatic motifs from three major nucleotide sequence altering mechanisms: somatic mutation (single and INDEL), RNA editing and SNP (Figure 2). These somatic motifs were organized, annotated and placed into SMDB for further usage. SMDB was designed using MySQL, and the web interface is constructed using PHP and JavaScript. All results in SMDB are based on GRCh38 human genome reference. The database can be categorized into three large categories: TF somatic motifs, RBP somatic motifs and miRNA-related somatic motifs. miRNA-related somatic motifs were further divided into miRNA seeds and miRNA–mRNA 3UTR binding subsections. The queryable fields are genomic locations, motif type (gain or loss), motif sequence, project (available for ICGC and TCGA), mutation gene, motif gene, tissue type (available for GTEx), SNP ID, etc.

### Somatic motifs associated with TF binding sites

TFs usually bind to gene regulatory regions such as promoters. While the promoter is generally considered as a non-coding region, TF binding sites play critical roles in regulating gene transcription. The TCGA somatic mutation data were derived from exome sequencing data, thereby resulting in insufficient coverage in non-coding regions including promoters. Thus, TCGA somatic mutation data are not suitable for large-scale detection of TF binding somatic motifs. In contrast, the ICGC somatic mutation data consist of whole genome sequencing data generated from numerous projects. Therefore, we analyzed 78 700 582 ICGC somatic mutations to identify altered binding sequences for the 746 TF motifs extracted from JASPAR. The JASPAR TF motifs range from 6 to 15 nucleotides with an average length of 12 nucleotides. Our initial analysis revealed 35 408 somatic motifs (15 932 gains and 19 476 losses) distributed across the 746 TF motifs. However, some of the TF motifs listed in JASPAR are not located upstream of protein-coding genes and thus may not be functional in regulating gene expression. The top high-frequency somatic motifs are displayed in Table 1 as an example of SMDB. They include 9 mutations that likely create new motifs (i.e. gain of functions) and 12 mutations that potentially deactivate TF motifs (i.e. loss of functions). Nineteen of the 21 somatic motifs are attributed to INDELs, while the other 2 are caused by single point mutations. Insertions contribute to a large portion of these somatic motifs. For example, in the biliary tract cancer Singapore cohort (BTCA-SG), the high-frequency (18.31%) insertion of [CCCCTCCCCC]CTT at the upstream of *RCOR3* gene forms a new binding motif sequence C[CCCCCTCCCCC] for *ZNF148*, a TF related to multiple cancer risks by regulating *TERT*, an oncogene encoding the telomerase reverse transcriptase (16). Deletions can contribute to the elimination of a binding motif. Also in the BTCA-SG, at the upstream sequence of *CHARD19*, deletion of the sequence AAGGAACCCCCCACCGGGCCCCGCCCCTTACTC is observed in >18% of all tumors. This deletion removes the binding sequence GCCCCGCCCC for *KLF5*, a zinc finger TF that has previously been associated with multiple cancer types, including colon (25), breast cancer (26), esophageal cancer (27), etc. Our analysis also confirmed the well-established *TERT* promoter mutations that create new *ETS* protein-binding motifs. In the skin cancer Australia cohort (MELA-AU), three gained *ETS* binding motifs (i.e. TTCCGG) were identified by our analysis, consistent with published data (7). Two of the three motifs occurred in the promoter region of *TERT*. The first one occurred at a frequency of 11.48% (Table 1), and the other occurred at a frequency of 9.84%. The functions of these mutations in driving *TERT* expression have been verified in human cancer cell lines (7,28,29). Intriguingly, our analysis revealed formation of the TTCCGG motif occurring in the promoter region of *RPS20*, which encodes a ribosomal protein. The frequency of this *RPS20* promotor mutation in melanomas is 14.75%, higher than the frequency of each single *TERT* mutation; however, it is currently unknown how *RPS20* promoter mutation affects its transcription.

**Table 1.** ICGC somatic motif analysis against JASPAR transcript binding factors

Project	Chr^a^	Location^b^	Gene (upstream distance)^c^	Mutation	Strand^d^	Types	Affected motif (TF)^e^	Frequency^f^
LMS-FR	8	18390725	NAT2 (dist = 557)	A>ATTA	+	Gain	[ATTA]AA (Arid3a)	11.94%
LMS-FR	8	18390725	NAT2 (dist = 557)	A>ATTA	+	Gain	GTC[ATTA]A (HOXC4)	11.94%
BTCA-SG	9	93095871	CARD19 (dist = 346)	AAGGAACCCCCCACCGGGCCCCGCCCCTTACTCG>G	−	Loss	[GCCCCGCCCC] (KLF5)	18.31%
BTCA-SG	7	101166514	VGF (dist = 945)	C>CC	−	Loss	CCCA[C]CTGCGC (ZEB1)	12.68%
BTCA-SG	1	211259205	RCOR3 (dist = 72)	C>CCCCCTCCCCCCTT	+	Gain	C[CCCCCTCCCCC] (ZNF148)	18.31%
BTCA-SG	1	211259205	RCOR3 (dist = 72)	C>CCCCCTCCCCCCTT	+	Loss	[C]GCCCCTCCCC (MAZ)	18.31%
MELA_AU	8	56074582	RPS20 (dist = 10)	C>T	+	Gain	T[T]CCGG (ETS1)	14.75%
MELA_AU	5	1295135	TERT (dist = 67)	C>T	−	Gain	T[T]CCGG (ETS1)	11.48%
BTCA-SG	8	86514404	RMDN1 (dist = 31)	CA>C	+	Gain	CGCCC[C]TCCCC (MAZ)	12.68%
LMS-FR	5	116050856	ARL14EPL (dist = 610)	GTCTG>G	−	Gain	TGC[G]TG (ARNT)	16.42%
LMS-FR	19	43826512	ZNF283 (dist = 809)	T>GTT	−	Loss	TGCG[T]G (ARNT)	14.93%
LMS-FR	19	14476342	PTGER1 (dist = 988)	T>GTT	−	Loss	TGCG[T]G (ARNT)	13.43%
BTCA-SG	2	88056086	KRCC1 (dist = 303)	T>TA	+	Loss	A[T]TAAA (Arid3a)	14.08%
BTCA-SG	12	18090325	RERGL (dist = 132)	T>TA	+	Loss	T[T]TAAAAAAAAA (ZNF384)	11.27%
BTCA-SG	5	42812249	SELENOP (dist = 173)	T>TA	+	Loss	T[T]TAAAAAAAAA (ZNF384)	11.27%
BTCA-SG	17	81398932	BAHCC1 (dist = 442)	T>TA	+	Loss	A[T]TAAA (Arid3a)	11.27%
BTCA-SG	6	150599621	PLEKHG1 (dist = 264)	T>TA	+	Loss	A[T]TAAA (Arid3a)	11.27%
LMS-FR	20	63499644	EEF1A2 (dist = 561)	T>TTCCGGGT	−	Gain	[TTCCGG] (ETS1)	11.94%
LMS-FR	2	68953575	GKN2 (dist = 682)	TGAA>T	+	Gain	AT[T]AAA (Arid3a)	17.91%
LMS-FR	11	4998233	OR51L1 (dist = 750)	TGCGTGT>T	−	Loss	[TGCGTG] (ARNT)	10.45%
LMS-FR	11	4998231	OR51L1 (dist = 752)	TGCGTGTGT>T	−	Loss	[TGCGTG] (ARNT)	10.45%

^a^Chromosome.

^b^Genomic location in GRCh38.

^c^Mutation gene is the gene the somatic mutation occurred upstream to. The upstream distance is displayed in the parentheses.

^d^Strand where the somatic motif is observed: +, positive strand; −, negative strand.

^e^The actual somatic motif sequence; nucleotide in the brackets indicates the mutated position and nucleotide.

^f^Mutation frequency = number of subjects with mutation/total number of subjects.

SNPs may enhance binding efficiency if the minor allele creates a TF binding sequence (15). Our somatic motif analysis of dbSNP data identified 379 121 somatic motifs (175 126 gains and 203 995 losses). GTEx eQTL somatic motif analysis identified 3331 somatic motifs (1643 gains and 1688 losses). eQTL somatic motif analysis on TF binding motifs shows that of the 47 876 unique upstream eQTLs, 3311 (∼7%) caused gain or loss of TF binding motifs in JASPAR, which intuitively explains the SNP expression regulation mechanism.

### Somatic motifs associated with RBPs

Next, we examined somatic mutations, RNA editing and SNP’s effects on RBP motifs. We focused on RBP motifs using data in four major RBP databases [ATtRACT (21), oRNAment (22), RBPDB (23) and RBPmap (24)]. TCGA mutation-based somatic motif analysis identified 5 484 261 somatic motifs (2 617 624 gains and 2 866 637 losses) associated with RBPs. However, no somatic motif occurs with a frequency >5% in our analysis. ICGC mutation-based somatic motif analysis identified 384 755 937 RBP somatic motifs (195 947 274 gains and 188 808 663 losses).

dbSNP somatic motif analysis against RBP binding motifs revealed 1 478 913 372 somatic motifs (698 044 904 gains and 780 868 468 losses). GTEx *cis*-eQTL somatic motif analysis against RBP binding motifs identified 28 336 423 somatic motifs (14 050 215 gains and 14 286 208 losses). Noticeably, many of the top eQTLs are INDEL eQTLs rather than single nucleotide eQTLs. One of the interesting RBP target genes, *SELENOF*, a cancer-related gene in the folate metabolism pathway, has two intronic eQTLs that cause gain and loss of *NOVA1* binding motifs. Of the 2 066 133 unique eQTLs in 3′-UTRs and introns, 2 065 869 (99.99%) have RBP somatic motifs and 1 265 570 (61.25%) have the same eQTL target genes as the RBP target genes. The regulation mechanism of these eQTLs can be intuitively explained by the gain or loss of RBP motifs due to the SNPs.

Initial somatic motif analysis of REDIportal RNA editing on RBP binding motifs from the four RBP databases revealed 181 568 somatic RBP motifs (64 414 gains and 117 154 losses). One RNA editing event can cause simultaneously loss and gain of binding motifs in the same RBP. For example, RBP *ZFP35* has two binding motifs differentiated by one nucleotide (ACCTG[C] versus ACCTG[T]), according to the ATtRACT database. The RNA editing event on the reverse strand (T-to-C) at 3′-UTR regions of *BPNT1* changes the sequence ACCTG[T] to ACCTG[C], which causes both gain and loss of *ZFP35* motifs. Detailed results for RBP somatic motif analysis are not presented in the manuscript; they can be directly queried in SMDB.

### miRNA seed and target somatic motifs

miRNAs regulate mRNA through their seed sequences. Somatic mutations occurring in the seed regions can substantially alter the mRNA targets. With ICGC and TCGA somatic mutation data, our somatic motif analysis detected 135 and 418 altered miRNA seeds in ICGC and TCGA, respectively. However, the majority of these altered miRNA seeds are caused by singleton mutations and none of these showed a frequency >5%. TargetScan (30) was used to predict mRNA targets for the original and new seed sequences. On average, the difference between the original seed targets and somatic seed targets is 70% for ICGC and 72% for TCGA. These results show that somatic mutations in miRNA seeds can lead to a substantial mRNA target shift. The biological effects of such mRNA target alterations have been demonstrated by previous studies (5,9,10).

RNA editing in miRNA seed regions has been shown to have a substantial impact on target mRNA selection and silencing efficiency (31). By applying RNA editing events in 17 TCGA cancer types to the SomticMotif tool, we identified two somatic miRNA seeds. The A-to-I RNA editing event on position 63819626 of chromosome 9 caused miR-4477b seed TT[A]AGGA to become TT[G]AGGA, which affects ∼66% of mRNA targets according to TargetScan’s prediction. This editing event was observed in 11 out of 17 TCGA cancer types with RNA editing data. The editing frequency ranges from 4.23% to 91.36% by cancer type.

Target mRNA 3′-UTR binding sequences for the miRNA seeds were obtained from starBase 2.0 (20). Somatic motif analysis using TCGA mutation data revealed 453 927 altered miRNA–mRNA 3′-UTR binding sequences. Somatic motif analysis using ICGC mutation data revealed 560 370 altered miRNA–mRNA 3′-UTR binding sequences. All 20 top hits are caused by INDELs. Many of the top hits have already been studied with miRNAs in cancers. For example, the highest mutation frequency (37%) insertion of T>TTT at chromosome 9, position 122847654 in the leiomyosarcoma French cohort occurred in the 3′-UTR region of *RC3H2*, which was recently found to facilitate cell proliferation by targeting miRNA miR-101-3p in oral squamous cell carcinoma. The second top hit with a 27% mutation frequency in the same cohort occurred in 3′-UTR of *SRSF7*, which has been shown to be target of multiple miRNAs and causes splice variants in renal cancer cells (32).

Somatic motif analysis on miRNA–mRNA 3′-UTR binding sequences using dbSNP data and GTEx *cis*-eQTL data revealed 3 094 366 and 529 721 somatic motifs, respectively. Top 22 somatic miRNA–mRNA 3′-UTR binding sequences caused by eQTL were ubiquitous (observed in all 49 tissue sites in GTEx), single nucleotide eQTLs, and the eQTL target gene is the same as the 3′-UTR gene. Of the 71 713 unique 3′-UTR eQTLs, 11 020 (15.36%) have altered miRNA–mRNA 3′-UTR binding sequences, which may help explain the regulation effect of eQTLs. Somatic motif analysis on miRNA–mRNA 3′-UTR binding sequences using REDIportal RNA editing data identified 19 202 somatic motifs. Detailed results for miRNA somatic motif analysis are not presented in the manuscript; they can be directly queried in SMDB.

## CONCLUSION

The identification of altered binding motifs resulting from somatic mutations or RNA editing has imminent scientific benefits. A myriad of studies have been conducted based on independent cases of such somatic motifs. Nearly all of them focus on the gain of somatic motif. We presented the first thorough SMDB and it stands out by identifying both losses and gains of important somatic motifs. The cascading biological effect from gain of an important motif is relatively easier to observe than the effect of loss of a motif. Because a binding sequence may have many targets, losing one may not cause a strong detrimental effect. However, some transcriptional effects can still be detected. For example, somatic mutations in the *SDHD* promotor region disrupted *ETS* binding motif and significantly reduced *SDHD* gene expression (33). We also identified this loss of motif in *SDHD*, except that this mutation did not meet the >10% mutation frequency threshold. Analysis using real somatic mutation data, RNA editing data and SNP data from large consortiums revealed some well-known and millions of novel somatic motifs. Many of the novel somatic motifs are of high frequencies deserving follow-up studies to examine functional mechanisms in more detail. By conducting large-scale analysis, we show that while some of the well-known somatic motifs have been studied, plenty of high potency targets await validation. Those high-frequency targets are curated in our database SMDB for easy search, browsing and bulk downloading.

## DATA AVAILABILITY

The SMDB and the related resources can be accessed and downloaded at http://www.innovebioinfo.com/Database/SMDB/Introduction.php.
